# Complete Genome Sequence of Human Enterovirus Strain 71 (EV71/Taipei/3118/2011), Isolated from a Patient in Taiwan

**DOI:** 10.1128/genomeA.01375-14

**Published:** 2015-01-08

**Authors:** Chia-Pei Lin, Jiung-Liang Liu, Lung-Yuan Chen, Yi-Chao Liu, Hsiu-Chi Wang, Shih-Jie Lin, Pin-Chun Chen, Kun-Teng Wang, Chih-Hung Huang, Yi-Chan Yang, Hwei-Fang Cheng, Daniel Yang-Chih Shih, Der-Yuan Wang

**Affiliations:** a Food and Drug Administration, Ministry of Health and Welfare, Taipei, Republic of China (Taiwan); b Ministry of Health and Welfare, Taipei, Republic of China (Taiwan); c Institute of Biochemical and Biomedical Engineering, National Taipei University of Technology, Taipei, Republic of China (Taiwan)

## Abstract

This full-length genome sequence of human enterovirus strain 71 (EV71/Taipei/3118/2011) was isolated from a clinical patient in Taiwan in 2011. According to the phylogenetic analysis, the complete genome sequence in this study is part of the subgenotype C4.

## GENOME ANNOUNCEMENT

Human enterovirus strain 71 (EV71) has been determined to be part of the *Picornaviridae*, which cause mild and self-limiting hand, foot, and mouth disease (HFMD) in young children and infants under the age of 5 years. EV71 has caused epidemic HFMD predominantly in the Asia-Pacific region, such as Taiwan, China, Japan, Malaysia, Singapore, and even Australia. This virus has been associated with more severe cases such as aseptic meningitis, encephalitis, or even death (1). The genome of EV71 is about 7.4 kb, consisting of a 5′-untranslated region, P1 structure protein region, P2 and P3 functional protein regions, and a 3′-untranslated region containing a long poly-A tail. This virus can be further classified into three main genotypes (A, B, and C) and 11 subgenotypes (A, B1 to B5, and C1 to C5) (2). Understanding of the genotypes of EV71 strains in the epidemic regions is important for the development of novel strategies for the prevention and treatment of the diseases associated with EV71.

In this case, we obtained a human EV71 (EV71/Taipei/3118/2011) that was isolated at the Taiwan Centers for Disease Control (Taiwan CDC) in 2011. The clinical isolate was collected from a patient with a clinical diagnosis of a severe case of HFMD in Taiwan. The isolate was inoculated in African green monkey kidney cells (vero cells, ATCC CCL-81) and propagated up to three passages. EV71-infected cells were harvested and preserved as an EV71 master viral bank. After virus propagation, several synthetic oligonucleotide primer pairs based on the alignment of available genome sequences of different EV71 strains were designed to amplify overlapping fragments that span the whole genome of EV71. The full-length genome sequence of strain EV71/Taipei/3118/2011 was established by assembling overlapping fragments with the SeqMan program of the Lasergene 7 package (DNASTAR).

Nucleic acid and protein sequence alignments were analyzed by use of the ClustalW2 program (3). The viral genome sequence of the strain EV71/Taipei/3118/2011 was composed of 7,262 nucleotides (nts) including the poly(A) tail. The 5′-untranslated region was found to be 665 nts, followed by an open reading frame (ORF) excluding the structural protein region P1 (2,586 nt), the functional protein regions P2 (1,734 nt), P3 (2,259 nt), and the 3′-untranslated region (18 nt). The contents of A, U, G, and C were 27.2%, 24.9%, 23.8%, and 24.2%, with GC content of 48.0%. Phylogenetic trees were constructed by means of the neighbor-joining method with the use of MEGA software, version 6.0, to estimate the viral gene relationship with selected enterovirus strains obtained from GenBank. The results of phylogenetic analyses suggest that strain EV71/Taipei/3118/2011 belongs to subgenotype C4.

### Nucleotide sequence accession number.

This whole-genome sequence of EV71/Taipei/3118/2011 isolated in Taiwan in 2011 has been deposited in GenBank under the accession number KM593929.
